# Exploring intelligent hospital management mode based on artificial intelligence

**DOI:** 10.3389/fpubh.2023.1182329

**Published:** 2023-08-14

**Authors:** Dezhi Mi, Yong Li, Kangying Zhang, Chaoni Huang, Wenjia Shan, Jiangbo Zhang

**Affiliations:** ^1^General Office, Shanghai Municipal Hospital of Traditional Chinese Medicine, Shanghai, China; ^2^Shanghai Municipal Hospital of Traditional Chinese Medicine, Shanghai, China; ^3^Logistics Support Department, Shanghai Municipal Hospital of Traditional Chinese Medicine, Shanghai, China; ^4^Bidding Department, Shanghai Shenkang Health Capital Construction Administration Co., Ltd., Shanghai, China; ^5^Luban Software Co., Ltd., Luban Research Institute, Shanghai, China

**Keywords:** intelligent, hospitals, management mode, patients, artificial intelligence

## Abstract

**Objective:**

To address the challenges posed by the COVID-19 pandemic, our hospital developed an intelligent hospital management mode specifically tailored to COVID-19 patients.

**Methods:**

This study included patients with confirmed diagnosis of COVID-19 admitted to our hospital between January 2020 to December 2022. We sought to explore intelligent hospital management mode based on artificial intelligence (AI) and new technologies such as 5G, Internet of Things (IoT), wearable devices, robots, and small programs.

**Results:**

Intelligent hospital management mode can help improve the quality of life of patients, while also improving the management ability of the hospital, as it can automatically deliver timely patient reminders, maintain environmental cleanliness, and help in the transportation of medical equipment without any manual labor. This can help in conserving time and reducing the workload of medical staff. Moreover, itcan help intellectualize patient admission, patient discharge, and hospital management, thereby helping in providing efficient medical and patient humanistic care.

**Conclusions:**

The development of intelligent management mode can reduce the burden of medical personnel and the probability of developing infection and bring about timely and better patient care. Intelligent management can play a pivotal role in control of the epidemic, treating patients, allocation of resources, tracing the root cause of the virus, and monitoring.

## Introduction

The novel coronavirus (COVID-19) outbreak has been one of the most infectious disease events the world has faced in nearly a century (1). In 2022, the Omicron variant affected 29 Chinese provinces, resulting in more than 100 medium-high risk areas across the country (2). Currently, China is facing prevention and control challenges in curbing the spread of the Omicron variant. As the number of confirmed COVID-19 cases continues to rise, there is a shortage of medical resources (3). In particular, the number of beds for critically ill patients is seriously insufficient, resulting in many patients not being admitted to hospitals for isolation treatment (4). Currently, China is recording more 3000 cases per day with the available bed capacity of approximately of only 23000 in big provinces like Jilin. This could lead to the spread of the COVID-19 and subsequently emerging variants.

Makeshift hospitals play a pivotal role in isolating patients and cutting off transmission caused by social interaction (1–3). Makeshift hospitals are mobile medical spaces, generally composed of medical functional units, ward units, technical support units and other parts, with emergency treatment, surgical treatment, clinical testing and other functions. Therefore, in March 2022, the National Health Commission (NHC) required each province to build two to three hospitals to limit virus transmission (2, 3). However, the development of these kind of hospitals is faced with various challenges, such as providing security for medical staff, patients, and services, achieving more scientific and effective zoning control, improving the efficiency of medical and administrative staff, and minimizing the risk of transmission within hospitals (1, 4, 5). In order to deal with these challenges, new smart hospitals can be established, which utilize advanced technology and have improved construction, operation, management, and other aspects of hospitals.

Development of China's medical and health care has gone through various stages, including the establishment of free medical care and labor insurance (2), basic medical security for urban workers and residents (3), and formation of rural cooperative medical security (4, 5). A series of national policy documents reflect the importance and necessity of high-quality operation of public hospitals in the new era (6, 7). Driven by policies, information construction and the application of big data are increasingly important in supporting hospitals' high-quality operation and development (6, 7). Electronic medical records in the early stage of information construction can no longer meet the demands of efficient hospital management in the new era; hence, it is necessary to reorganize the in-depth development strategy, promote the construction of a hospital management mode and top-level framework of information based on big data (8) to improve efficiency and expand the development space.

**Figure 1:**
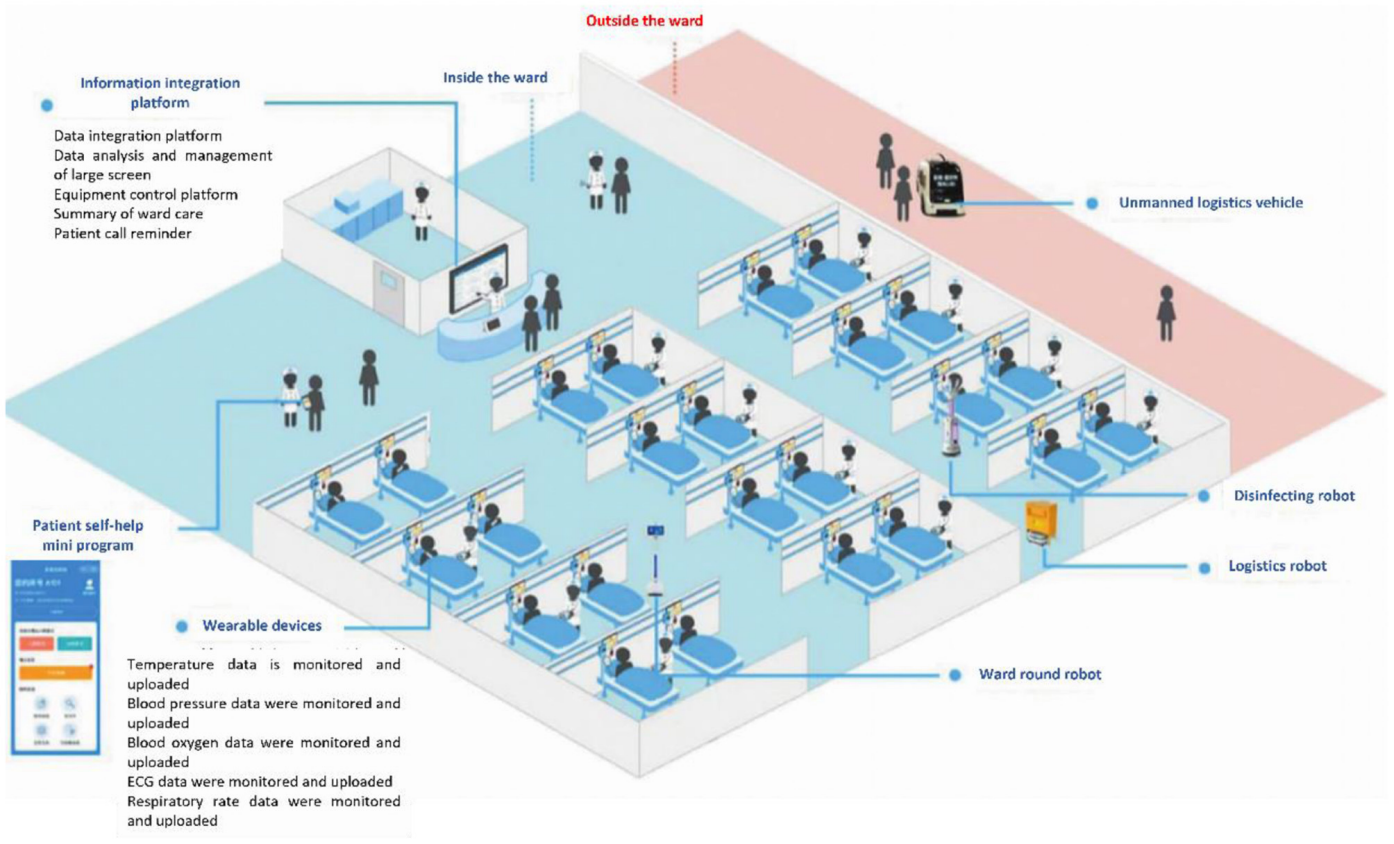
Schematic diagram of intelligent hospital management mode.

Therefore, this paper aims to explore the construction of an intelligent hospital management mode (Figure 1) based on AI and 5G, the Internet of things (IoT), wearable devices, robots, small programs, and other new technologies.

## Methods

### Design, sample and time period

This retrospective cohort study was conducted on COVID-19 patients admitted to our hospital between January 2020 and December 2022.

### Inclusion criterion

Patients were included in this study if (1) they have confirmed diagnosis of COVID-19 infection via PCR assay, (2) were adherent and cooperative to treatment, and (3) they provided written consent to participate in this study.

### Exclusion criterion

Patients were excluded from this study if (1) they have any significant comorbidities or (2) they did not consent to take part in this study.

### Equipment and technology

This studyexplored and constructed an intelligent hospital management mode based on artificial intelligence (AI) and new technologies such as 5G, Internet of Things (IoT), wearable devices, robots, and small programs.

## Results

### Content of intelligent hospital management mode construction

#### Role of intelligent devices in nursing

**(a) Ward round robots**: Ward round robots have automatic navigation and obstacle avoidance functions. They can form real-time high-definition multi-point video connections and are equipped with various functional equipment and accessories to integrate existing healthcare systems. Doctors can remotely access patients' medical records and examination data and images under the auxiliary support of ward round robots, helping them in remote multidisciplinary consultation and providing them with remote disease notifications. The ward round robots can work for 8–12 h on a single charge. It has various sensors and accessories, including 10 m lidar, infrared, ultrasonic and voice modules, Kinect, RealSense, an 8-megapixel camera, a 13.3-inch display, a 5G network, and SLAM algorithm (9). Using which ward round robots can accurately perform various functions like automatic navigation, obstacle avoidance, route intelligent planning, human-computer interaction, and wireless video image transmission. Doctors can easily access the information a medical robots collect using mobile phone applications. Thus, robots can substitute doctors in makeshift cabins to complete ward rounds, provide guidance and communicate with patients. Medical staff can carry out medical activities for patients without being physically present, thereby improving the efficiency of medical service response. Ward round robots can also act as the convergence center of smart handheld medical devices in the ward. Through wireless connection to smart handheld medical devices, it can integrate multiple diagnostic parameters, such as auscultation, electrocardiography (ECG), blood pressure, and body temperatures. Using this technology, experts outside the ward can remotely monitor heart rate and auscultate, which can assist them in reaching a diagnosis and effectively treating patients. This forms the basis of remote bedside rounds and consultations.

**Figure 2:**
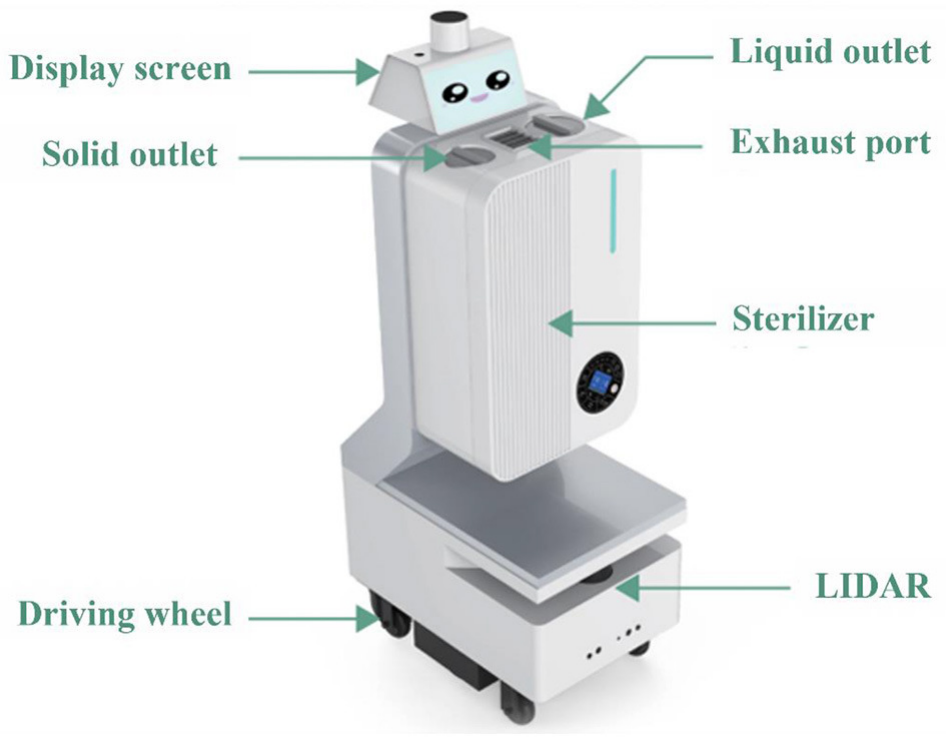
Disinfection robot.

**(b) Sterilizing robots**: Sterilization of hospitals has been challenging in the current outbreak caused by a mutant strain of Omicron, as manual disinfection may not be sufficient for effective sterilization. Hence, sterilization robots or disinfection robots can be utilized to ensure automated sterilization without any time limits for operation. Disinfection robots first estimate the total disinfection time according to the space to be covered. Then, it carries out disinfection at 360 degrees without a dead angle. Disinfection robots can carry a maximum of 40 kg of disinfectant and run for 4–12 h (depending on different disinfection modes) per 1-h charge time. Disinfection robots comprise three modes: ultraviolet, ultra-dry fog, and air filtration. (10). Unlike artificial spraying, ultra-dry mist spraying can disperse to form aerosol particles, helping in better diffusion of disinfectants. Disinfection robots also can monitor disinfectant concentration in the air in real-time. This allows for effective disinfection while also ensuring the safety of doctors from harmful levels of disinfectants. Disinfection robot can automatically sense the disinfection route and time customized program reservation. They are equipped with various intelligent systems, such as automatic indoor autonomous navigation, autonomous return charging, intelligent obstacle avoidance function, and autonomous ladder. Moreover, the robot has multiple safety protection technologies, such as far-field slowdown, near-field emergency stop, pedestrian first walk, and mechanical collision avoidance. The stable and unimpeded operation guarantees the machine's and personnel's safety. Furthermore, disinfection robots are intelligent disinfection equipment that integrates autonomous positioning and navigation, multi-mode disinfection, friendly man-machine interaction, intelligent scheduling, and management. With the help of their nano-level atomizing disinfection and multi—angle UV lamp disinfection function, Disinfection robots provide a comprehensive and efficient disinfection and sterilization guarantee for the air environment in the ward. Hospital waste is a major public health concern as improper medical waste management can have serious health and environmental consequences, such as radiation burns, sharps-inflicted injuries, and toxic exposure to pharmaceutical products such as mercury and dioxin that have adverse consequences on the exposed individuals. In this context, disinfection robots can manage ecofriendly disposal of hospital waste. Before disposal, disinfection robots can treat waste items with the help of disinfectants and steam sterilization, such as autoclaves to kill pathogens. This can help reduce both potential public health threats and environmental damage (see Figure 2).

**Figure 3:**
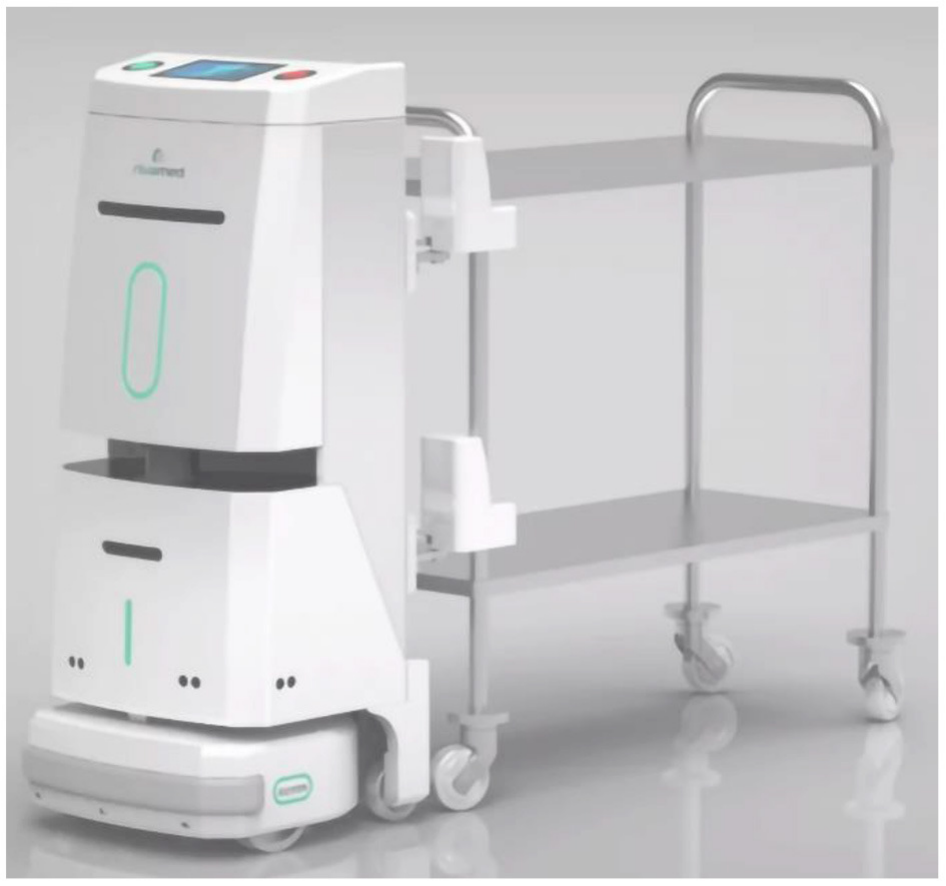
Logistics robot.

**(c) Logistics robot**: The increased workload of the distribution of supplies, meals, consumables, and medical waste in the hospital is a major problem (11), especially for the outbreak and surge of COVID-19 outbreak. Therefore, hospitals must adopt a variety of intelligent logistics robots for each large ward, which can greatly reduce the working pressure on medical staff and effectively reduce the hidden danger of cross-infection. Intelligent delivery robots can be used for hospital medicine, meals, surgical clothing, high-value consumables, and other supplies distribution, thereby supporting the box modulator customization to meet the specific needs of hospital departments. Logistic robots can carry a maximum load of up to 500 kg. One robot is equivalent to four full-time delivery personnel in terms of self-service and unmanned distribution of medical supplies. Moreover, it has the ability to function 24 h non-stop, potentially reducing the simple repetitive labor time of medical staff, which may be utilized in higher-value work. The intelligent logistics robot is safe and reliable, able to store and issue whole-process records with strict authority control. It can accurately identify the type and dosage of drug access, help monitor the whole drug administration process, and reduce the risk of theft of consumables and drugs. Logistic robots are also equipped with special functional medicine cabinets, which allow for the strict control of consumables and drugs while providing suitable storage conditions (see Figure 3).

### Smart wearable devices

**Figure 4:**
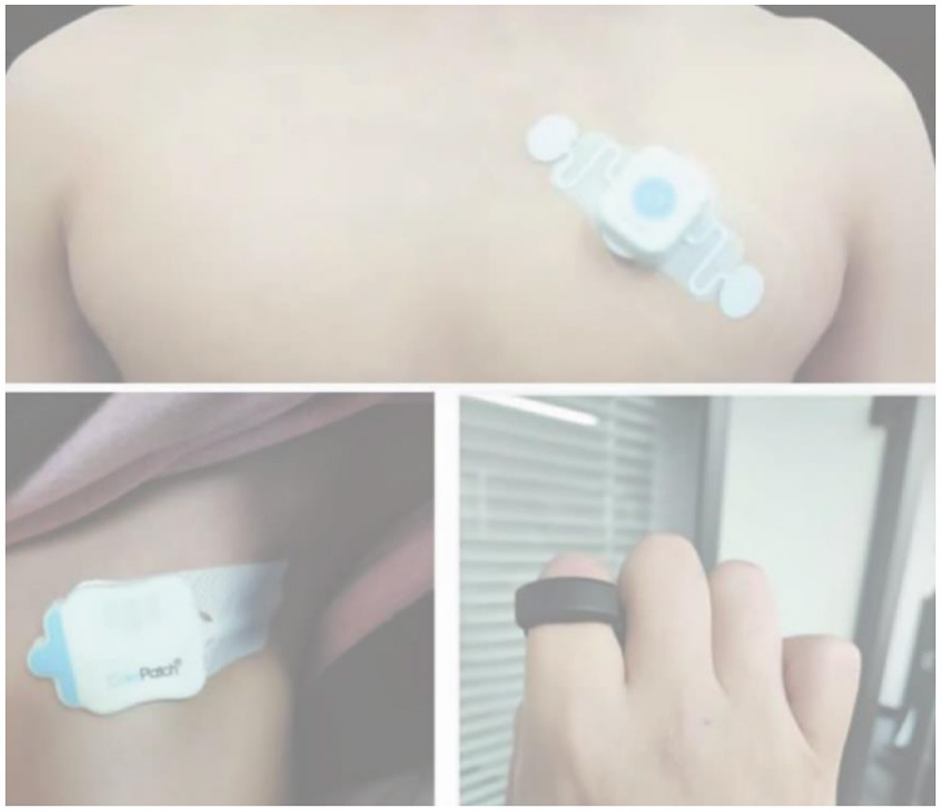
Wearable devices.

Manual measurement of vital signs data such as body temperature and ECG of patients every day is time-consuming, especially when the doctor-patient ratio in the hospital is very low. Also, measuring a patient's body temperature, heart rate, respiratory frequency, blood oxygen saturation, and other data daily leads to increased occupational exposure, increasing the risk of infection transmission to medical staff. Moreover, manual vitals measurement may also cause unnecessary delays in the initiation of the management of patients and cause abnormal measurements to be missed. To overcome these issues, wearable devices (see Figure 4) can be used to monitor patients' vital signs in real-time. Wearable devices can reduce the frequency of nurses walking back and forth and the time consumed during manual measurements. In addition, with its ability to automatically input various vital signs data, it can help in reducing the error rate during manual input, detecting changes in patient's vital signs in the cabin in real-time, and quickly identifying abnormal signs, thereby assisting doctors in reaching fast and accurate diagnosis so that the timely treatment for patients with abnormal signs can be initiated. It involves data transmission to the cloud to provide intelligent analysis and help improve prevention and control efficiency (12). The adopted wearable devices have the following features: (1) Self-defined high and low-temperature alarm value and an automatic reminder of high and low temperature; (2) continuous use of a charge for seven days, allowing for 24/7 continuous monitoring; (3) measuring accuracy of +0.1°C; (4) net weight of about 7g, fully flexible bionic curve design, easy to wear and take off.

### Network aspects of the hospital intelligent management infiltration

The 5G medical network system enables user terminals to conduct data queries, operations and other basic services on the public and private domains. Compared with traditional Wi-Fi networks, 5G high-quality wireless networks can help achieve better data access, better integration with the inherent mobile network, and more stable and strong anti-interference data transmission. 5G network can ensure high-speed, stable and safe data transmission to large number of users with the help of various intelligent systems like loT equipment, robotic scheduling and video communication.

**Figure 5:**
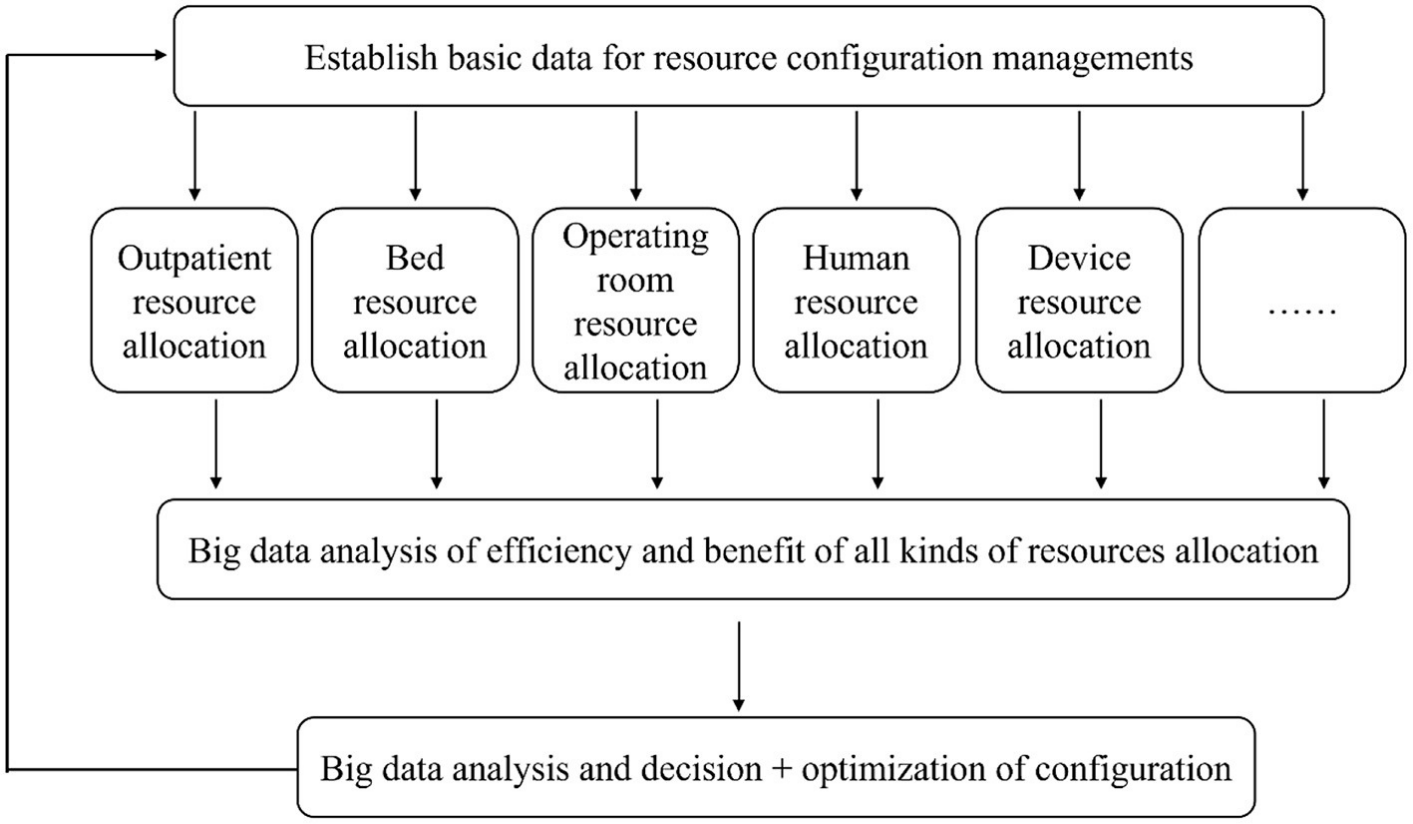
Big data-based decisions to optimize resource allocation.

Big data security governance's objective primarily focuses on micro subjects and their high-frequency interactions, helping explore correlation and find similarities among subjects. (2). Due to its large volume of data processing, fast speed of information extraction, and strong stability of prediction results, big data has been widely used in the prevention and control of COVID-19. For the treatment of patients in hospitals, big data can predict the trend of the epidemic prospectively through the formation of data sets and AI-based algorithm models, which can then help the government determine the regions and groups in need of medical resources, governments in decision-making, and optimizing the treatment process and resource allocation. By analyzing the big data on process efficiency and combining it with the actual business and resource allocation, the management model can be optimized, and the process efficiency can be dynamically detected (Figure 5).

Artificial intelligence technology has been widely used in medical diagnosis and the establishment of smart makeshift hospitals. A large number of real-time patient temperature, heart rate, ECG and oxygen saturation data can be accumulated using wearable devices. Moreover, using AI deep learning technology, diagnosis of various diseases can be reached, such as ventricular contraction (single, paired, double, triple, ventricular tachycardia), atrial early (single, paired, double, triple, atrial tachycardia), bradycardia/tachycardia, diseases with long RR intervals (interphase, atrial fibrillation/atrial flutter, and atrioventricular block). AI algorithms applied to disease monitoring, and diagnosis can help determine the condition and shorten the time of diagnosis. Big data and artificial intelligence algorithms and technologies support many internet applications, which are an indispensable part of intelligent management (13).

The establishment of intelligent management mode hospitals has certain requirements that must be fulfilled, such as IoT medical and health applications, wearable IoT devices for remote data collection and transmission of data regarding patient physical signs, mobile ward round and nursing, identification of medical supplies, management of medical materials, treatment of medical waste, supervision of large medical equipment and medical robots (14). Intelligent management has played an important role in everything from equipment personnel positioning to operation management. These intelligent applications reduce the burden of medical staff and can effectively alleviate the problem of medical system overload while also providing an important means for hospital management.

Robots are electromechanical products controlled by computers, which carry out repeated imitation in work and complete complex and repetitive work (15). Intelligent robots are equipped with sensing systems that can be used for monitoring, intelligent pattern recognition, deep learning, and autonomous decision-making functions. Even in complex environments, intelligent robots can complete various tasks through intelligent behaviors (16), simulating human intelligence. Intelligent robots comprehensively utilize mathematical models, applied statistics, computer science, and operations research. The mapping, navigation, and obstacle avoidance technology based on multi-sensor fusion can meet the requirements of working in makeshift hospitals. Additionally, intelligent robots are playing various roles, from self-service ward rounds to cloud-based intelligent cleaning, disinfection, patrolling, and even delivering meals and medicine to patients, thereby reducing the workload of medical care and maximizing the efficiency of limited medical care resources.

## Discussion

### The application effect of intelligent hospital management mode

Wearable intelligent medical devices can sense non-sensory parameters, such as body temperature, heart rate, blood pressure, and blood oxygen. With remote wireless monitoring technology, doctors can note the vital signs of infected patients more quickly and thus carry out medical workup more efficiently and effectively. Additionally, reduced exposure of medical professionals to infected individuals also reduces the risk of infectious transmissions. The emergence of the patient self-help mini program has simplified the functions of personnel statistics, personnel trajectory, access control in relevant areas, thereby reducing the work burden of medical staff. Using various robots has previously been shown to save human and material resources. Disinfection robots can timely eliminate toxins from the environment, reduce the risk of virus transmission, and workload of medical staff. Robots also help deliver food, drinks, and medicine. Since robots do not require protective equipment, it can help conserve scarce materials like protective clothing, and masks. Robots can facilitate healthcare workers by making their work more efficient and safer. It is noteworthy to mention that in case of an unprecedented emergency that may lead to loss of power, hospital based on intelligent mode will require emergency and standby power systems that will act to provide Emergency Power Supply Systems (EPSS). The purpose of EPSS will be to provide continued power for critical life support systems and infrastructure regardless of any unprecedented circumstances.

ECG monitoring and unmanned distribution application supported by 5G technology can also provide valuable support for establishing smart shelters (17, 18). With the support of the 5G network, patients will be more satisfied with their medical experience and encounter decreased disease-associated anxiety. Using 5G high-quality and safe wireless network to achieve data access, 5G data stable transmission, strong anti-interference based on the breakthrough of the traditional Wi-Fi network to the bottleneck of data transmission and achieve integration with the inherent mobile network. In the construction of intelligent management mode of the hospital, 5G network can support the network use needs of tens of thousands of patients, and ensure the high-speed, stable, and safe transmission of data such as Internet of Things equipment, robot scheduling and video communication. Effective implementation of clinical pathways can standardize medical behaviors, reduce the possibility of human-induced cross-infection, reduce costs, and improve treatment effects in preventing and controlling infectious diseases (19, 20).

### Construction and development status of intelligent hospital management

Chinese public hospital operation management has various business complexes, and the information management needs of each are different. Barriers to the linkage of business management are reflected in the following aspects: (i) lack of materials, equipment, logistics management and basic guarantee management system, (ii) lack of standardization of the databases, and (iii) variabilities in the information level of each security management system (21), making it difficult to achieve system interconnection among businesses in the face of different management requirements. In addition, the comprehensive operation management of the hospital lacks the application of global monitoring, which makes it difficult to achieve efficient management and avoid the blind area of management. Currently, there is an urgent need to break the “information island” and eliminate the “information chimney,” which can be achieved with the construction and development of intelligent hospital management (22).

### Purpose and idea of intelligent hospital management mode

Intelligent hospital management mode has the potential to improve efficiency through scientific epidemic prevention and control, which can be attained by adopting an online multi-code integrated intelligent program (mobile APP) to automatically associate patient information. This includes nucleic acid detection, vaccination, intelligent analysis, and comprehensive judgment. Intelligent hospital management mode is based on the concept of “real-time and big data monitoring,” which can be achieved with the help of IoT and AI algorithms. Based on this, medical staff can conduct comprehensive real-time analysis of the entire medical record content, including diagnosis, symptoms, examination, and test results, helping them to respond quickly to diseases. In the intelligent hospital management mode, robot technology replaces medical staff to perform ward rounds, disinfection, and transportation, thereby improving the efficiency of medical care and reducing the risk of infection transmission to healthcare workers. The small program technology provides comprehensive coverage to patients, including admission, medical service, daily information push, ward navigation, materials distribution, sterilization, and report inquiry. This helps medical staff save time in reaching diagnosis and administering treatment, thus enabling more accurate patient monitoring and management. Therefore, in the new era, integrity and collaboration in establishing an intelligent hospital management mode are key to the high-quality operation of hospitals. Effective implementation of intelligent management mode in hospitals warrants scientific planning and top-level design to ensure forward-looking and systematic cognition (23).

### Limitations

While intelligent hospital management mode has the potential to promote the development and application of AI in healthcare, it also faces certain challenges. These include the need for continuous technological innovation to meet evolving medical practice requirements, as well as concerns about the acceptability of new technologies among healthcare workers. Incorporating of AI technologies also faces privacy concerns. Implementation of AI requires a big, representative data set. However, larger data sets are limited by patient privacy and data exchange. Therefore, new privacy and data management principles are necessary to train algorithms on these datasets while also maintaining individual's privacy by encoding individual's data through various stages of encryption. Additionally, at the level of current treatment practice for hospitalized patients, the data formats obtained by different wearable devices have not been completely unified, and the statistical dimensions of data adopted by various data caliber (such as 5G and ward round robot) are still different. At the same time, cross-institutional and cross-hospital data flow are insufficient, and data urgently efficient integration.

### Improvements and future directions

1. Epidemic prevention and control have provided more scenarios for applying various digital technologies. In the future, the intelligent hospital management mode, technologies, and products such as the IoT 5G, AI, robots, and Web mini programs will be further utilized to create an integrated and intelligent platform for prevention and control. Promoting publicity and communication of new technologies and constantly creating application scenarios of new technologies can encourage healthcare workers to make better use of these innovative applications. Therefore, to support and improve joint epidemic prevention and control, active use of 5G, cloud computing, big data, and other technologies should be encouraged.
2. To improve data quality, relevant institutions and hospitals should work together to strengthen data governance ability. In addition, it is also necessary to unify data standards and calibrate and define data sources, data formats, data management requirements, and data processing processes. In order to ensure the authenticity of data, full use of the existing big data platform and big data construction achievements can help achieve data convergence and integration in a timely and effective manner. Furthermore, it is also necessary to actively promote cross-hospital cooperation and exchanges and expand the standardization and application boundaries of new technologies.

## Conclusion

The establishment of an intelligent hospital management mode is essential for fighting against COVID-19, as it can play an important role in preventing and treating the infection. Hospitals that use intelligent management mode have more requirements than ordinary hospitals. In the intelligent management mode, all kinds of information systems must be planned and built simultaneously including integrating intelligent technologies such as the IoT, 5G, AI, robots, mini-Web programs, and various wearable medical devices. The construction of intelligent management mode can reduce the burden of medical personnel and the probability of developing infection and bring about timely and better patient care. Intelligent management can play a pivotal role in control of the epidemic, treating patients, allocation of resources, tracing the root cause of the virus, and monitoring. Since the introduction of smart healthcare, mature concepts and systems have emerged. However, there is still plenty of room for advancement that warrants future studies in this domain. There are several areas where future studies can help improve the implementation of intelligent-based hospital mode in clinical practice. Future studies can help accelerate the maturity and stability of the technologies described through upgrades. Moreover, implementing automated EHR systems can also be explored as it can increase productivity and continuously update as more data are available. To ensure patients' privacy and to provide more secure and reliable data transmission, techniques such as blockchain can also be investigated by future studies.

## Data availability statement

The original contributions presented in the study are included in the article/supplementary material, further inquiries can be directed to the corresponding author.

## Author contributions

DM contributed to the conception and design of the study. YL acquired the data. KZ and CH performed the data analysis and wrote the first draft of the manuscript. WS and JZ revised the manuscript critically. All authors contributed to manuscript revision, read, and approved the submitted version.
